# Botanical Substitution in Traditional ‘Rang Chuet’ Preparations: Distinguishing the Potential Xenobiotic Modulation of *Thunbergia laurifolia* Lindl. from the Hepatotoxic Risks of *Crotalaria spectabilis* Roth

**DOI:** 10.3390/ijms27188389

**Published:** 2026-09-20

**Authors:** Kitti Leesiam, Arunporn Itharat, Weerachai Pipatrattanaseree, Puritat Kanokkangsadal, Thishnapha Vudhironarit, Vilailak Tiyao, Sunita Makchuchit, Pranporn Kuropakornpong, Neal M. Davies

**Affiliations:** 1 Graduate School, Faculty of Medicine, Thammasat University, Klongluang, Pathumthani 12120, Thailand; 2 Department of Applied Thai Traditional Medicine, Faculty of Medicine, Thammasat University, Klongluang, Pathumthani 12120, Thailand; 3 Center of Excellence in Applied Thai Traditional Medicine Research (CEATMR), Thammasat University, Klongluang, Pathumthani 12120, Thailand; 4 Regional Medical Science Center 12 Songkhla, Ministry of Public Health, Mueang Songkhla, Songkhla 90100, Thailand; 5 Department of Pharmacology, Phramongkutklao College of Medicine, Ratchathewi, Bangkok 10400, Thailand; 6 Faculty of Pharmacy and Pharmaceutical Sciences, Katz Group-Rexall Centre for Pharmacy and Health Research, University of Alberta, Edmonton, AB T6G 2E1, Canada; ndavies@ualberta.ca

**Keywords:** Rang Chuet, detoxification, ethnopharmacology, molecular docking

## Abstract

Rang Chuet is a traditional Thai medicinal plant widely used for detoxification, although the name is applied to several botanically distinct species. This study investigated the ethnopharmacological basis and potential molecular mechanisms underlying this practice by integrating practitioner knowledge, botanical authentication, phytochemical profiling, and molecular docking. Structured interviews were conducted with 30 experienced traditional medicine practitioners from five regions of Thailand to document the therapeutic use and preparation of Rang Chuet. Botanical identification confirmed three species marketed under this name: *Crotalaria spectabilis* Roth, *Curcuma rangjued* Saensouk & Boonma, and *Thunbergia laurifolia* Lindl. Most practitioners used Rang Chuet primarily for detoxification, with *C. spectabilis* and *T. laurifolia* being the predominant species. LC–MS/MS analysis identified fifteen phytochemicals, including compounds common to both species, such as apigenin, quercetin, and caffeic acid, together with species-specific constituents including rosmarinic acid and α-spinasterol in *T. laurifolia*. Molecular docking suggested that selected phytochemicals may interact with xenobiotic-sensing receptors, with α-spinasterol showing favorable binding to the Pregnane X Receptor (PXR) and apigenin and catechin to the Aryl Hydrocarbon Receptor (AhR). However, *C. spectabilis* is a recognised source of hepatotoxic pyrrolizidine alkaloids, and because molecular docking cannot distinguish agonist from antagonist activity or establish functional receptor activation, these predicted interactions should not be interpreted as evidence of detoxification efficacy or safety. These findings provide a scientific framework linking traditional knowledge, botanical diversity, phytochemical composition, and potential receptor-mediated mechanisms, and highlight the public health risk of botanical substitution under the shared vernacular name “Rang Chuet.” These findings are hypothesis-generating and warrant future functional and toxicological validation, particularly regarding *C. spectabilis*.

## 1. Introduction

Detoxification is a complex physiological process that occurs primarily in the liver, with additional contributions from the intestines, kidneys, lungs, and skin. It comprises a coordinated series of enzymatic reactions, broadly described as Phase I (functionalisation), Phase II (conjugation), and Phase III (transport and excretion), that convert lipophilic xenobiotics and endogenous toxic metabolites into less toxic, water-soluble compounds that can be readily eliminated from the body [1,2]. Because xenobiotic metabolism is frequently accompanied by increased oxidative stress, endogenous antioxidant defences also play an important protective role during detoxification [3].

Ethnopharmacology provides an essential bridge between traditional knowledge and modern biomedical research by documenting how medicinal plants are selected, prepared, and used within indigenous healthcare systems. International consensus guidelines emphasize that ethnopharmacological investigations should integrate rigorous botanical identification, standardized interview methodologies, comprehensive documentation of traditional knowledge, and appropriate scientific validation to ensure reproducibility and cultural integrity [4]. Such studies not only preserve traditional medical knowledge but also facilitate the discovery of novel therapeutic agents and improve understanding of the relationships between traditional use, phytochemical composition, and pharmacological activity.

Rang Chuet (*Thunbergia laurifolia* Lindl., Acanthaceae) is included in Thailand’s National List of Essential Medicines as a traditional herbal medicine indicated for detoxification. It has traditionally been used for the treatment of poisoning caused by pesticides, alcohol, heavy metals, poisonous foods, environmental contaminants, and various toxic substances, as well as for fever, inflammation, liver disorders, and allergic diseases [5,6]. However, caution has been recommended in patients with diabetes mellitus because of its reported hypoglycaemic activity, while its mild diuretic and laxative effects may influence the pharmacokinetics of concomitantly administered medications [6]. Phytochemical investigations have demonstrated that *T. laurifolia* contains a wide range of biologically active secondary metabolites including flavonoids, phenolic acids, caffeic acid derivatives, rosmarinic acid, iridoid glycosides, lignans, and other polyphenolic compounds [5,7]. Many of these constituents possess antioxidants, anti-inflammatory, hepatoprotective, antimicrobial, immunomodulatory, and cytoprotective activities [8,9]. Extracts of *T. laurifolia* have also been shown to induce Phase II detoxification enzymes, regulate cytochrome P450 expression, modulate P-glycoprotein activity, and reduce oxidative stress, thereby providing mechanistic support for its traditional detoxifying properties [7,10].

Despite this pharmacological potential, the vernacular name Rang Chuet does not correspond to a single botanical species. Traditional textbooks and practitioners recognise more than one plant under this name, creating uncertainty about which species should be used for detoxification and complicating both clinical practice and the scientific evaluation of this herbal medicine. Traditional practitioners use several different plants under the same name, yet no study has systematically determined which species are actually used, how their use differs across practitioners and regions, or whether their phytochemical composition explains their shared and divergent therapeutic reputations. To address this gap, an ethnopharmacological survey was conducted among traditional herbal medicine practitioners from the five regions of Thailand to document the traditional use of plants collectively known as Rang Chuet and to determine the botanical species corresponding to this vernacular name. The phytochemical constituents of the two Rang Chuet species most frequently used by practitioners were then characterised by LC-MS/MS, and the identified compounds were subjected to molecular docking analysis to generate mechanistic hypotheses regarding their interactions with molecular targets involved in detoxification pathways. This study is the first to integrate ethnopharmacological documentation, botanical authentication, phytochemical profiling, and molecular docking to explain why multiple botanically distinct species are traditionally recognised under the single vernacular name Rang Chuet, thereby providing scientific evidence in support of its traditional use and mechanistic insight into its potential detoxifying properties.

This study therefore aimed to: (1) identify which plant species are referred to as Rang Chuet throughout Thailand; (2) document their traditional medicinal uses; (3) characterise their phytochemical profiles; and (4) investigate potential detoxification mechanisms using molecular docking.

## 2. Results

### 2.1. Ethnopharmacological Survey Findings

**Figure 1:**
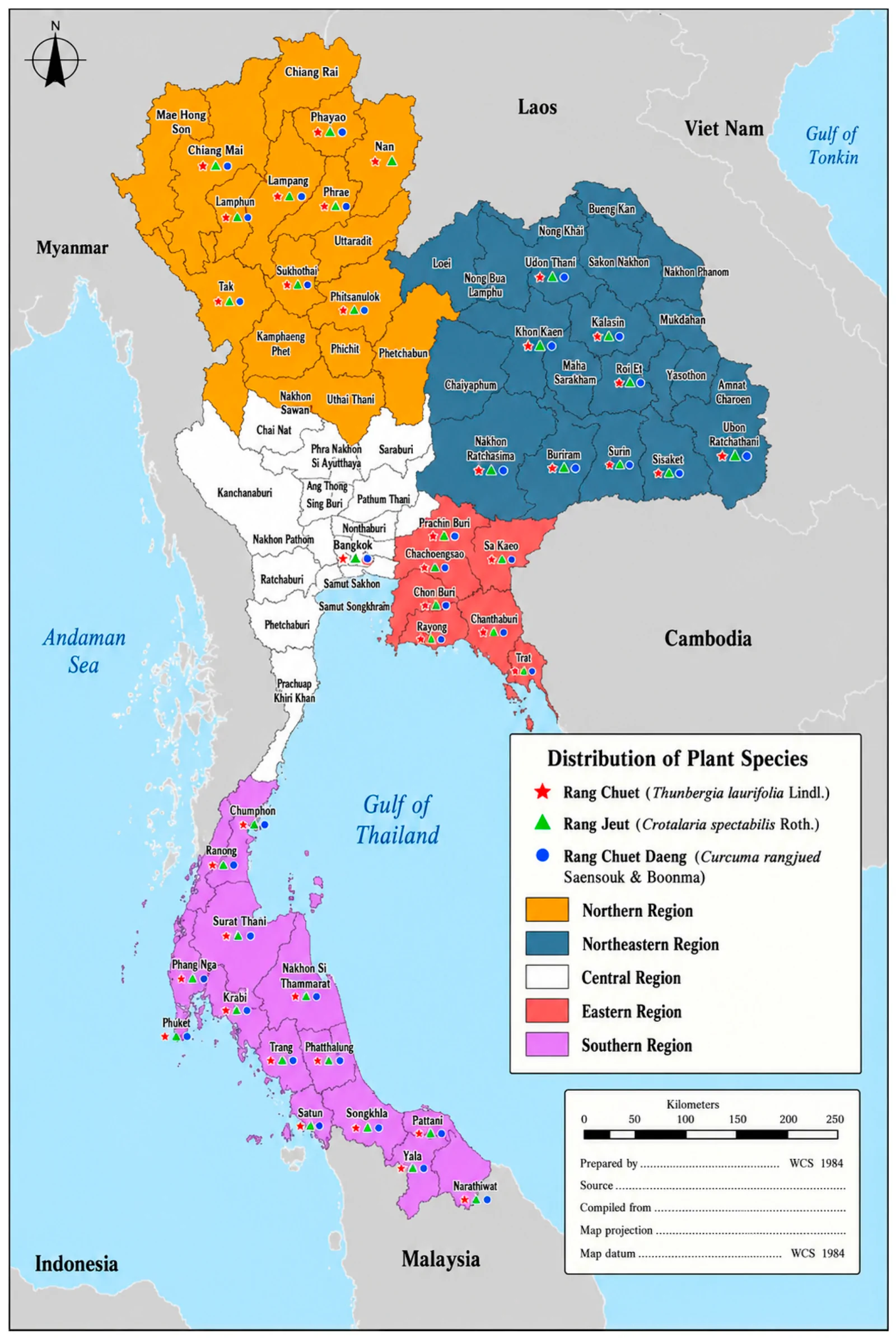
Geographical Distribution of Rang Chuet Species Used in Thailand.

From in-depth interviews using a structured questionnaire to document their knowledge, therapeutic applications, and preparation methods of plants known as Rang Chuet, the 30 practitioners from 5 regions of Thailand were recruited for this study (Figure 1). They had clinical experience with more than 10 years. Most participants were male, with a mean age of 68 years. Approximately 87% had an educational level below a bachelor’s degree. Regarding their primary occupation, 40% practiced herbal medicine, while another 40% were engaged in agriculture. The major areas of expertise included herbal medicine (83.3%), followed by traditional massage (20.0%) and bone-setting (16.7%). More than half of the participants (53.3%) did not possess an official medical license, and 56.7% had over 30 years of clinical experience. All participants reported using Rang Chuet primarily as a detoxifying agent. Approximately 30% used only Rang Chuet Tao, while another 30% reported using 3 different plant species collectively known as Rang Chuet. Single-herb preparations were more commonly employed than polyherbal formulations. The 40% of traditional folk usually used *Thunbergia laurifolia* Lindl. or Rang Chuet for detoxification as a medication ingredient (Table S1).

Several practitioners noted that the choice of Rang Chuet species is not arbitrary but reflects regional plant availability and locally transmitted teaching lineages: practitioners in the northern and north-eastern regions more frequently reported using Rang Chuet Tao (*T. laurifolia*), whereas those in the central region more often reported using Rang Chuet Ton (*C. spectabilis*) or mixed preparations. When asked why different botanical species are accepted under a single name, practitioners generally explained that all three plants are believed to share a common detoxifying “virtue” passed down through oral tradition, and that substitution among Rang Chuet species is considered acceptable practice when a preferred species is locally unavailable. Several senior practitioners traced their knowledge of Rang Chuet to teachers who themselves reported multi-generational use of the name for more than one plant, suggesting that the botanical ambiguity long predates its documentation in modern Thai herbal references. This shared traditional rationale, rather than simple taxonomic confusion, may explain why the vernacular grouping has persisted despite the species’ clear botanical distinctiveness.

The ethnopharmacological findings indicated that the knowledge on the use of Rang Chuet herbal medicine revealed that 30% of all herbal preparations utilize the Rang Chuet vine species, and that 30% employ all three species, namely Rang Chuet vine, Rang Chuet tree (Rang Chuet ton), and Rang Chuet tuber. All herbal medicine used Rang Chuet Tao for the detoxification of various toxic substances. In addition, these plants were incorporated into polyherbal formulations for the management of diabetes mellitus and hypertension, as well as for postpartum care to control hemorrhage and facilitate the elimination of lochia. Regarding plant parts used, the tuber of Rang Chuet was the most commonly utilized part (43.3%) For Rang Chuet tree, the root was predominantly used (33.3%) For the Rang Chuet vine, the leaves were the most frequently used part (43.3%). In addition, 100% of the herbal preparations utilize the Rang Chuet vine species, while the purple-flowered variety is used in 100% of cases. All Rang Chuet herbal preparations are used for antidotal purposes, including detoxification and toxin cleansing. In terms of mode of use, the majority of preparations consist of single-herb usage (56.7%). See Figure 1 and Table 1.

**Figure 2:**
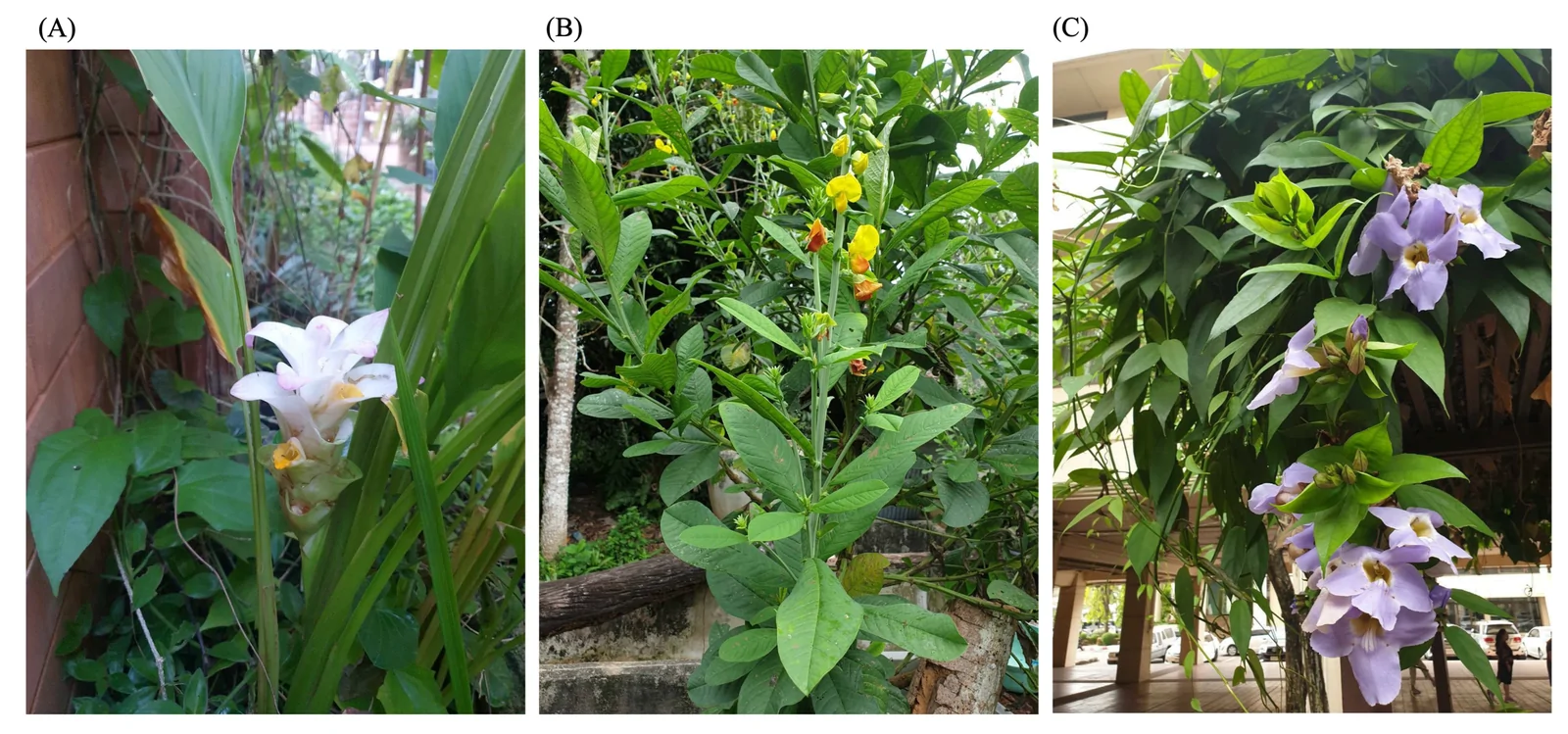
Three Species of Thai plant name “Rang Chuet” (**A**) *Curcuma rangjued* Saensouk & Boonma (**B**) *Crotalaria spectabilis* Roth (**C**) *Thunbergia laurifolia* Lindl.

Botanical identification confirmed that three species of Rang Chuet were *Crotalaria spectabilis* Roth (Fabaceae) or Rang Chuet Ton, *Curcuma rangjued* Saensouk & Boonma (Zingiberaceae) or Rang Chued Hua, *Thunbergia laurifolia* Lindl. (Acanthaceae) or Rang Chuet Tao (Figure 2). Two Rang Chuet species, as the most commonly used for detoxification were *Crotalaria spectabilis* Roth and *Thunbergia laurifolia* Lindl. LC–MS/MS analysis successfully identified fifteen phytochemical compounds with previously reported pharmacological activities. The ubiquitous occurrence of apigenin, quercetin, and caffeic acid, together with species-specific constituents such as rosmarinic acid and α-spinasterol, provides a phytochemical basis for their potential biological activities in Section 2.2.

### 2.2. Molecular Docking Studies

Identified Phytochemicals in *Crotalaria spectabilis* and *Thunbergia laurifolia* Extracts by LC-MS/MS Several compounds demonstrated widespread distribution across both species and multiple plant parts (Table 2). Apigenin and caffeic acid were consistently identified in the leaf, stem, and root extracts of both *C. spectabilis* and *T. laurifolia*, suggesting their ubiquitous presence in these Rang chuet species (Figure S1a). Similarly, quercetin was found across all tested parts (leaf, stem, and root) of both species. Conversely, some compounds exhibited more restricted distributions. Rosmarinic acid was detected exclusively in the leaf, stem, and root extracts of *T. laurifolia*, indicating its species-specific presence. α-Spinasterol was also found only in *T. laurifolia*, specifically in its leaf and root extracts. Catechin showed a limited presence, being identified only in the leaf extracts of both *C. spectabilis* and *T. lauriforia* (Figure S1b).

The exclusive occurrence of rosmarinic acid in *T. laurifolia* may help explain why this species has historically become the officially recognised medicinal species despite the continued traditional use of other Rang Chuet species. Because α-spinasterol was likewise detected only in *T. laurifolia* and, as described below, subsequently showed the strongest predicted PXR interaction in the docking analysis, its restricted distribution may itself form part of the phytochemical basis for this species’ pre-eminent traditional and regulatory status, while the constituents shared with *C. spectabilis* (apigenin, quercetin, caffeic acid) may account for the common detoxifying reputation attributed to both plants under the Rang Chuet name.

#### 2.2.1. Binding Affinities of Ligands with PXR (PDB ID: 1M13)

The predicted binding affinities for the eight compounds from *Crotalaria spectabilis* Roth and *Thunbergia laurifolia* Lindl. and control ligands with the Orphan Nuclear Receptor PXR (PDB ID: 1M13) are summarized in Table 3. The co-crystallized ligand hyperforin, a known PXR agonist, showed a binding affinity of −9.53 kcal/mol. Similarly, the known PXR antagonist ketoconazole showed a binding affinity of −9.70 kcal/mol.

The compounds from *Thunbergia laurifolia* Lindl., α-spinasterol demonstrated the most favorable binding affinity to PXR at −10.13 kcal/mol, which is notably lower (indicating stronger binding) than that of the co-crystallized agonist, hyperforin. This suggests α-spinasterol shows binding characteristics consistent with a potential PXR agonist. Rosmarinic acid, Rutin, and Apigenin also exhibited a strong binding affinity of −8.20, −9.70, and −8.06 kcal/mol, suggesting that both compounds may interact favorably with the PXR binding site. Other compounds, including Quercetin (−7.86 kcal/mol), Catechin (−7.83 kcal/mol), Gallic acid (−5.93 kcal/mol), and Caffeic acid (−6.06 kcal/mol), showed moderate binding affinities to PXR.

#### 2.2.2. Binding Affinities of Ligands with AhR (PDB ID: 8XS9)

Table 3 also presents the predicted binding affinities of the selected compounds with the bHLH-PAS transcription factor Aryl hydrocarbon receptor (AhR, PDB ID: 8XS9). The co-crystallized ligand, beta-Naphthoflavone (AhR agonist), showed a strong binding affinity of −12.23 kcal/mol. The known AhR antagonist, Ilantimod, exhibited a binding affinity of −4.93 kcal/mol.

Among the compounds from *Crotalaria spectabilis* Roth and *Thunbergia laurifolia* Lindl., apigenin and catechin exhibited the most favorable predicted binding affinity toward AhR at −9.50 kcal/mol. Other compounds with relatively strong binding affinities included quercetin −8.70 kcal/mol) and caffeic acid (−8.00 kcal/mol). Moreover, gallic acid and rosmarinic acid demonstrated a moderate binding affinity to AhR with −6.90 and −7.30 kcal/mol, respectively. In the meantime, rutin and α-spinasterol showed mild interaction with AhR of −4.83 and −5.40 kcal/mol binding affinity, respectively.

#### 2.2.3. Two-Dimensional (2D) Interaction Analysis with PXR

**Figure 3:**
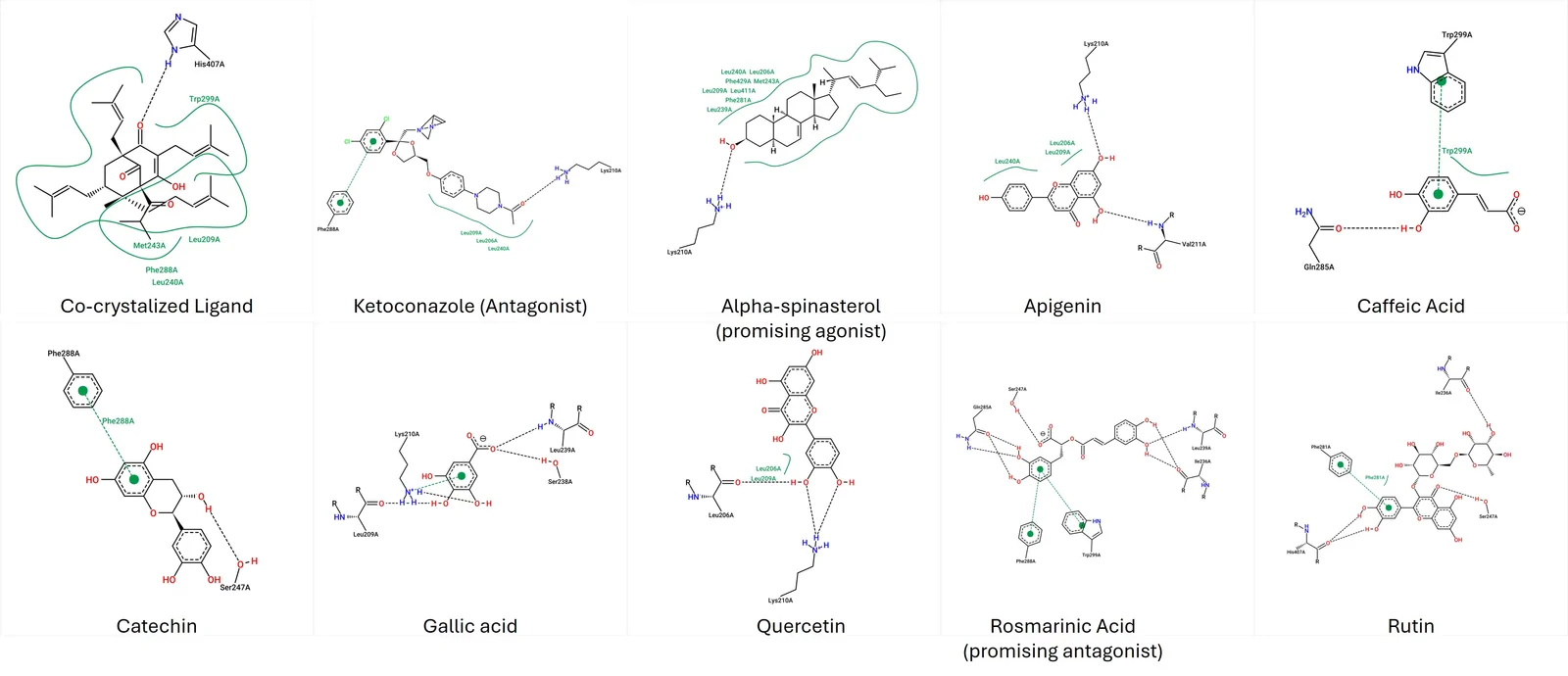
2D Interaction diagrams of selected ligands with PXR (PDB ID: 1M13).

The detailed molecular interactions between selected ligands and the PXR binding pocket are depicted in the 2D interaction diagrams (Figure 3). The co-crystallized ligand, hyperforin, was observed to form hydrophobic interactions with residues such as Met243A, Leu209A, Phe288A, Leu240A, and Trp299A, along with a hydrogen bond with His407A. α-spinasterol, predicted as a favourable PXR ligand, predominantly engaged in extensive hydrophobic interactions with several hydrophobic residues including Leu240A, Leu206A, Leu209A, Leu411A, Phe429A, Met243A, Phe281A, and Leu239A. It also formed a hydrogen bond with Lys210A. This robust network of interactions likely contributes to its strong binding affinity. Rosmarinic acid, a promising antagonist, formed a hydrogen bond with Gln285A and exhibited pi-stacking interactions with Phe288A and Trp299A. Ketoconazole, a known PXR antagonist, also formed hydrogen bonds with Lys210A and showed hydrophobic contacts with Leu209A, Leu206A, Leu240A, and pi-stacking with Phe288A.

Other compounds from *Crotalaria spectabilis* Roth and *Thunbergia laurifolia* Lindl. also displayed specific interactions within the PXR binding site: Apigenin formed hydrogen bonds with Ser247A and His407A, along with pi-stacking interactions with Phe281A. Catechin showed hydrogen bonds with Ser247A and hydrophobic interactions with Phe288A. Gallic acid engaged in hydrogen bonds with Lys210A, Ser238A, and Leu239A, complemented by pi-stacking with Leu209A. Quercetin formed hydrogen bonds with Lys210A and displayed hydrophobic interactions with Leu206A and Leu209A. Rutin formed hydrogen bonds with Ile236A and Ser247A, and pi-stacking with Phe281A. Caffeic acid engaged in hydrogen bonds with Ile236A, Leu239A, Phe288A, Ser247A, and Trp299A, with pi-stacking also observed with Phe288A and Trp299A.

#### 2.2.4. Two-Dimensional (2D) Interaction Analysis with AhR

**Figure 4:**
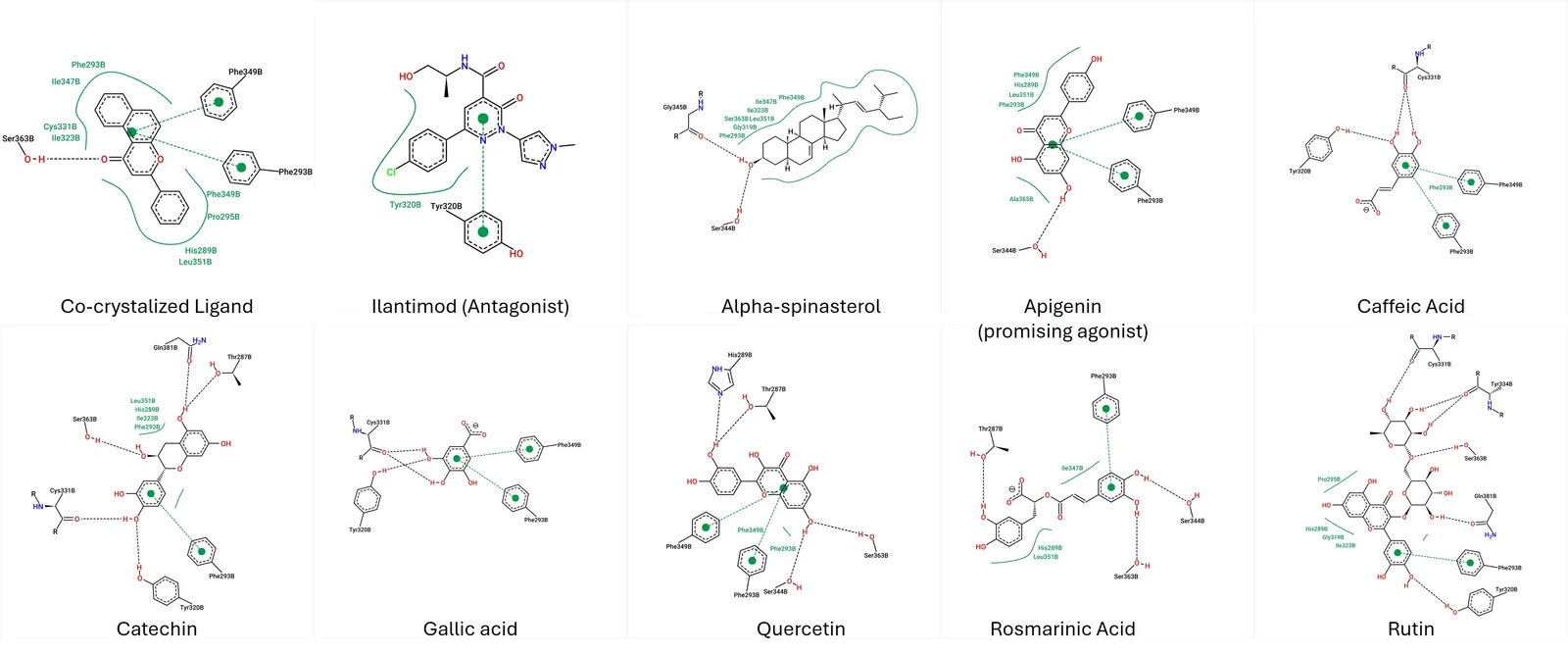
2D Interaction diagrams of selected ligands with AhR (PDB ID: 8XS9).

The molecular interactions between selected ligands and the AhR binding pocket are presented in the 2D interaction diagrams (Figure 4). The co-crystallized ligand, beta-Naphthoflavone, formed hydrophobic interactions with Phe293B, Phe349B, Pro295B, His289B, Leu351B, and Cys331B, as well as a hydrogen bond with Ser363B.

Apigenin, predicted as a favourable AhR ligand, exhibited key interactions including a hydrogen bond with Ser344B and hydrophobic contacts with Phe293B, Phe349B, His289B, and Leu351B. This Comprehensive set of interactions aligns with its favorable binding affinity.

The known AhR antagonist, Ilantimod, displayed hydrophobic interactions with Tyr320B and a pi-stacking interaction with Tyr320B. α-spinasterol, which had an unfavorable binding affinity, showed a hydrogen bond with Ser344B and hydrophobic interactions with Gly345B, Phe293B, Ile347B, Phe349B, Ser363B, Ile323B, Gly319B, and Leu351B.

Other Rang Chuet compounds also contributed to varied interaction profiles within the AhR binding site: Catechin formed hydrogen bonds with Gln381B, Cys331B, Tyr334B, and Ser363B, along with hydrophobic interactions with Pro295B, His289B, Gly319B, and Ile323B. Gallic acid engaged in hydrogen bonds with Cys331B and Tyr320B, supplemented by pi-stacking interactions with Phe293B and Phe349B. Quercetin formed multiple hydrogen bonds with Thr287B, Ser344B, and Ser363B, and hydrophobic contacts with Phe293B, Phe349B, His289B, Leu351B, Ile323B, and Cys331B. Rosmarinic acid demonstrated hydrogen bonds with Ser344B, Ser363B, and Thr287B, along with pi-stacking with Phe293B, Ile347B, His289B, and Leu351B. Rutin, showing the highest (least favorable) binding affinity, engaged in hydrogen bonds with Cys331B, Tyr320B, Ser344B, and Ser363B, and hydrophobic interactions with Phe293B and Phe349B. Caffeic acid formed hydrogen bonds with Cys331B and Tyr320B, with pi-stacking interactions involving Phe293B and Phe349B.

## 3. Discussion

The present study represents one of the first comprehensive investigations integrating ethnopharmacological documentation, phytochemical characterization by LC–MS/MS, and molecular docking analysis to investigate the traditional Thai detoxification medicine known collectively as Rang Chuet. Rather than focusing solely on *Thunbergia laurifolia* Lindl., this study recognizes that the vernacular name “Rang Chuet” encompasses multiple botanical species that are used interchangeably or in combination by traditional practitioners. This distinction is important because botanical identity directly influences phytochemical composition, pharmacological activity, safety, and ultimately therapeutic efficacy. By combining field-based ethnopharmacological surveys with modern analytical chemistry and computational pharmacology, the present study provides a more comprehensive scientific framework for understanding the traditional use of Rang Chuet as a detoxifying herbal medicine.

### 3.1. Ethnopharmacological Significance of Rang Chuet in Thai Traditional Medicine

Traditional medical knowledge has served as the foundation for the discovery of numerous modern pharmaceuticals and continues to provide an invaluable resource for identifying medicinal plants with therapeutic potential [11,12,13]. In Thailand, medicinal plants remain widely incorporated into primary healthcare, particularly in rural communities where traditional healers preserve knowledge that has been transmitted over many generations. Documentation of this knowledge is increasingly important because rapid urbanization, changing lifestyles, and the declining number of traditional practitioners threaten the preservation of ethnopharmacological information [11].

The ethnopharmacological survey demonstrated remarkable consistency among traditional practitioners regarding the principal therapeutic application of Rang Chuet. All respondents identified the plants as detoxifying medicines, indicating a strong consensus across geographically diverse regions of Thailand. Such complete agreement is unusual in ethnobotanical investigations and suggests that detoxification represents the defining therapeutic indication of Rang Chuet within Thai traditional medicine. These findings are consistent with previous reports describing *Thunbergia laurifolia* Lindl. as one of Thailand’s most important medicinal plants for treating poisoning associated with pesticides, alcohol, toxic foods, heavy metals, environmental contaminants, and various chemical toxicants [5,6,14]. Experimental studies demonstrating hepatoprotective activity, antioxidant effects, induction of Phase II detoxification enzymes, and regulation of xenobiotic metabolism further support these traditional uses [7,8,9].

An important observation from the present survey was that traditional practitioners did not regard Rang Chuet as a single botanical species. Instead, three distinct medicinal plants—*Thunbergia laurifolia* Lindl., *Crotalaria spectabilis* Roth, and the tuberous form referred to locally as Wan Rang Chuet—were recognized as legitimate sources of Rang Chuet herbal medicine. Approximately one-third of practitioners reported using only *T. laurifolia*, whereas an equal proportion routinely incorporated all three species into therapeutic preparations. This finding highlights the complexity of traditional herbal medicine, where vernacular names frequently represent groups of medicinal plants sharing similar therapeutic indications rather than a single taxonomically defined species.

The use of multiple botanical species under a common vernacular name has important implications for both scientific research and herbal medicine quality control. Botanical substitution or variation can substantially influence phytochemical composition, pharmacological activity, efficacy, and safety, thereby contributing to inconsistent therapeutic outcomes. Similar taxonomic ambiguity has been documented for numerous medicinal plants throughout Southeast Asia and represents a significant challenge for the standardization of herbal medicines [11,12]. Consequently, rigorous botanical authentication and voucher specimen deposition should be regarded as essential components of ethnopharmacological investigations to ensure reproducibility and facilitate meaningful comparisons between studies.

The survey also revealed substantial variation in the plant parts selected for medicinal use. While the leaves of *T. laurifolia* were the most frequently utilized plant material, roots and stems were also commonly employed, and tubers constituted the principal medicinal component of Wan Rang Chuet. Such variation is pharmacologically relevant because secondary metabolite accumulation differs considerably among plant organs owing to differences in physiological function, environmental exposure, and developmental regulation. Leaves typically accumulate higher concentrations of flavonoids and phenolic antioxidants that protect against ultraviolet radiation and oxidative stress, whereas roots often contain distinct classes of metabolites associated with plant defense and rhizosphere interactions [15,16]. Consequently, variation in plant part selection may result in substantial differences in phytochemical composition and biological activity among traditional preparations.

Preparation methods documented during the survey further emphasize the diversity of traditional medicinal practices. Decoctions, infusions, fresh plant preparations, and ground herbal pastes were all commonly reported, either as single-herb remedies or as components of more complex polyherbal formulations. Extraction methodology is well recognized to influence the recovery of bioactive phytochemicals, particularly thermolabile flavonoids, phenolic acids, and glycosylated metabolites [11]. Differences in solvent polarity, extraction temperature, duration, and plant processing can therefore alter both the qualitative and quantitative phytochemical composition of herbal preparations, potentially affecting therapeutic efficacy. These observations underscore the need for standardized preparation methods when evaluating medicinal plants under experimental conditions.

Interestingly, every practitioner surveyed identified the purple-flowered variety of *T. laurifolia* as the preferred medicinal form. Although white-flowered variants have occasionally been reported, none of the respondents recognized these as therapeutically equivalent. This unanimous preference suggests that traditional practitioners distinguish medicinal plant varieties using morphological characteristics that may reflect underlying phytochemical differences. Whether flower color is associated with genetic variation, metabolite accumulation, or medicinal efficacy remains unknown and represents an interesting avenue for future phytochemical and genomic investigations.

Collectively, the ethnopharmacological findings demonstrate that traditional Thai knowledge concerning Rang Chuet is both sophisticated and internally consistent. Rather than representing a simplistic herbal remedy, Rang Chuet appears to constitute a complex medicinal system in which practitioners recognize multiple botanical species, select different plant organs according to traditional practice, and employ various preparation methods tailored to clinical circumstances. These observations reinforce the importance of ethnopharmacological research as a means of preserving traditional knowledge while simultaneously generating hypotheses that can be investigated using modern analytical and pharmacological techniques.

The integration of ethnopharmacological surveys with phytochemical profiling and computational pharmacology represents one of the principal strengths of the present study. Contemporary ethnopharmacology increasingly advocates multidisciplinary approaches that combine traditional knowledge with analytical chemistry, molecular biology, and systems pharmacology to elucidate mechanisms underlying herbal medicines [11]. Such approaches not only facilitate scientific validation of traditional medical practices but also support the development of standardized botanical medicines capable of meeting modern quality, safety, and efficacy requirements. The present work therefore contributes not only to understanding the traditional use of Rang Chuet but also establishes an evidence-based framework for future pharmacological and clinical investigations.

### 3.2. Phytochemical Characterization of Rang Chuet Species by LC–MS/MS

One of the major strengths of the present investigation was the integration of ethnopharmacological observations with comprehensive phytochemical characterization using LC–MS/MS. While traditional knowledge identifies plants based upon their therapeutic properties, phytochemical profiling provides the molecular basis for understanding these medicinal activities. High-resolution LC–MS/MS has become one of the most powerful analytical tools for investigating medicinal plants because it enables the rapid identification of structurally diverse metabolites within complex botanical extracts while minimizing the need for extensive compound isolation [16,17,18]. Consequently, metabolomic profiling not only supports botanical authentication but also facilitates the identification of bioactive constituents that may contribute to pharmacological activity.

The LC–MS/MS analysis identified fifteen phytochemicals distributed among the leaf, stem, and root extracts of *Thunbergia laurifolia* Lindl. and *Crotalaria spectabilis* Roth. These metabolites represented several important classes of natural products, including flavonoids, flavonoid glycosides, phenolic acids, phytosterols, chlorophyll derivatives, carotenoids, and polyphenolic compounds. Such chemical diversity is consistent with previous reports describing *T. laurifolia* as a chemically rich medicinal plant possessing multiple pharmacologically active secondary metabolites [5,6,7]. Rather than relying on a single active constituent, the medicinal properties of Rang Chuet are therefore likely to result from the synergistic or additive actions of numerous bioactive compounds acting simultaneously on multiple molecular targets.

The widespread occurrence of apigenin, caffeic acid, and quercetin throughout all examined tissues of both plant species suggests that these compounds represent conserved phytochemical markers of Rang Chuet. Their consistent detection in leaves, stems, and roots indicates that these metabolites participate in fundamental physiological functions within the plants and may provide a common biochemical basis for the shared traditional use of these species as detoxifying medicines. Flavonoids such as apigenin and quercetin are widely recognized for their antioxidant, anti-inflammatory, hepatoprotective, antimicrobial, and immunomodulatory activities, whereas caffeic acid contributes potent free radical scavenging activity together with modulation of inflammatory signaling pathways [15,19,20]. Their ubiquitous distribution across both botanical species therefore provides a plausible explanation for why traditional practitioners consider these plants therapeutically interchangeable despite belonging to different botanical families.

Among the compounds identified, quercetin deserves particular attention because it has been extensively investigated as one of the most biologically active naturally occurring flavonoids. Quercetin exhibits broad pharmacological activity including antioxidant, anti-inflammatory, antiviral, cardioprotective, neuroprotective, and hepatoprotective effects [21]. At the molecular level, quercetin regulates multiple intracellular signaling pathways, suppresses activation of NF-κB, inhibits production of pro-inflammatory cytokines, enhances endogenous antioxidant defenses, and modulates xenobiotic metabolism through interactions with cytochrome P450 enzymes and other detoxification pathways. These diverse biological properties make quercetin an attractive candidate for contributing to the traditional detoxification activity attributed to Rang Chuet.

Apigenin, another flavonoid detected consistently throughout both species, has similarly been reported to possess antioxidant, anti-inflammatory, anticancer, and hepatoprotective properties. In addition to directly scavenging reactive oxygen species, apigenin modulates apoptosis, inflammatory signaling, and cellular stress responses through regulation of MAPK, PI3K/Akt, NF-κB, and Nrf2 pathways. Previous studies have also demonstrated that apigenin influences xenobiotic metabolism through interactions with nuclear receptors and cytochrome P450 enzymes, thereby supporting its potential role in regulating detoxification processes [15,20]. The consistent occurrence of apigenin in both *T. laurifolia* and *C. spectabilis* further strengthens the hypothesis that flavonoids represent one of the principal classes of compounds responsible for the pharmacological activity of Rang Chuet.

Caffeic acid was likewise identified throughout all examined tissues of both species. Phenolic acids such as caffeic acid constitute an important component of plant antioxidant defense systems and have been widely investigated for their ability to reduce oxidative stress, inhibit lipid peroxidation, suppress inflammatory mediators, and protect tissues from toxic injury. Since oxidative stress is a common mechanism underlying chemical toxicity, the presence of caffeic acid provides an additional mechanistic explanation for the long-standing use of Rang Chuet as a detoxifying medicinal plant. Together with flavonoids, caffeic acid may contribute to maintaining intracellular redox balance while reducing the oxidative damage associated with xenobiotic metabolism [19].

In contrast to these universally distributed metabolites, several compounds demonstrated species-specific localization that may distinguish the pharmacological properties of *T. laurifolia* from those of *C. spectabilis*. Most notably, rosmarinic acid was detected exclusively in *T. laurifolia*, where it occurred in leaves, stems, and roots but was absent from *C. spectabilis*. This finding agrees with previous phytochemical investigations identifying rosmarinic acid as one of the characteristic phenolic constituents of *T. laurifolia* [5,7]. Rosmarinic acid possesses exceptionally strong antioxidant and anti-inflammatory activity and has been shown to reduce reactive oxygen species formation, inhibit complement activation, suppress inflammatory cytokine production, and protect tissues against chemically induced injury [22]. Its species-specific occurrence may therefore partially explain why *T. laurifolia* has received considerably greater attention in pharmacological studies than other Rang Chuet species.

Another notable finding was the exclusive detection of α-spinasterol in *T. laurifolia*. Phytosterols are increasingly recognized as biologically active plant constituents capable of modulating inflammation, oxidative stress, membrane stability, and lipid metabolism. Although α-spinasterol has received comparatively less attention than common phytosterols such as β-sitosterol, previous studies have reported anti-inflammatory, immunomodulatory, neuroprotective, and hepatoprotective activities. The identification of α-spinasterol only within *T. laurifolia* became particularly significant when considered alongside the molecular docking results, where it demonstrated the strongest predicted interaction with the Pregnane X Receptor (PXR). This observation suggests that α-spinasterol may represent one of the principal molecular mediators responsible for regulating xenobiotic metabolism within this species.

Catechin also exhibited a restricted distribution, being detected only in leaf extracts of both botanical species. This localization is biologically plausible because flavan-3-ols are commonly synthesized in photosynthetically active tissues where they protect against ultraviolet radiation and oxidative stress. Catechins possess potent antioxidant properties through direct scavenging of reactive oxygen species together with modulation of endogenous antioxidant enzymes and inflammatory signaling pathways. Although present only in leaves, their high biological activity suggests that they may contribute disproportionately to the medicinal value of leaf-based herbal preparations.

Several additional metabolites, including rutin, isoquercetin, lutein, chlorophyll derivatives, pheophytin, pheophorbide, hydroxypheophytin, and gallic acid, further illustrate the remarkable chemical diversity of the two Rang Chuet species. Glycosylated flavonoids such as rutin and isoquercetin may serve as important storage forms that undergo hydrolysis following ingestion, releasing their corresponding aglycones with enhanced biological activity. Likewise, chlorophyll degradation products have increasingly been recognized as possessing antioxidant, anti-inflammatory, and photoprotective properties rather than representing merely inactive degradation products. The coexistence of these diverse metabolite classes suggests that multiple phytochemical pathways collectively contribute to the biological activity of Rang Chuet rather than any single “active principle”.

An important implication of the present phytochemical findings concerns quality control and standardization of herbal medicines. Considerable variation in metabolite composition between botanical species, plant organs, harvest conditions, and preparation methods may substantially influence therapeutic efficacy. Because traditional practitioners utilize different species and plant parts under the common name Rang Chuet, standardization based solely on vernacular nomenclature may be inadequate. Instead, authentication should incorporate botanical identification together with chemical fingerprinting using validated analytical techniques such as LC–MS/MS. The identification of conserved metabolites, including apigenin, quercetin, and caffeic acid, together with species-specific markers such as rosmarinic acid and α-spinasterol, provides a valuable foundation for developing future quality control standards.

Overall, the LC–MS/MS analysis confirms that Rang Chuet is chemically complex and that its traditional medicinal properties are likely mediated through multiple bioactive constituents acting on complementary molecular pathways. Such phytochemical complexity is characteristic of many traditional herbal medicines and supports the concept of multi-component, multi-target pharmacology rather than single-compound therapeutic mechanisms. These findings also establish an important foundation for interpreting the molecular docking results, where several of the identified metabolites demonstrated favorable interactions with nuclear receptors regulating xenobiotic metabolism and detoxification.

### 3.3. Molecular Docking Analysis and Regulation of Xenobiotic Detoxification Pathways

Although ethnopharmacological knowledge and phytochemical characterization provide important evidence supporting the traditional use of medicinal plants, they do not by themselves explain the molecular mechanisms responsible for the observed therapeutic effects. Molecular docking has therefore emerged as an important computational tool for predicting interactions between phytochemicals and biologically relevant protein targets involved in disease processes and xenobiotic metabolism. Unlike conventional pharmacological assays that require purified compounds and extensive biological testing, molecular docking permits rapid screening of multiple naturally occurring compounds against selected receptors, thereby generating mechanistic hypotheses that can subsequently be validated experimentally [23,24]. Although docking cannot demonstrate biological activity directly, it provides valuable information regarding potential ligand binding, receptor selectivity, and structure-activity relationships.

The present study represents one of the first investigations to evaluate phytochemicals identified from *Thunbergia laurifolia* Lindl. and *Crotalaria spectabilis* Roth against two of the principal transcriptional regulators of xenobiotic metabolism, the Pregnane X Receptor (PXR) and the Aryl Hydrocarbon Receptor (AhR) These receptors function as molecular sensors that detect structurally diverse endogenous metabolites and environmental xenobiotics, subsequently regulating the expression of numerous enzymes and transport proteins responsible for detoxification. Activation of these receptors coordinates cellular responses that increase metabolism, conjugation, transport, and elimination of potentially harmful compounds while simultaneously protecting tissues from oxidative injury and inflammation [25,26,27].

The predicted binding affinities observed in the present study demonstrated clear differences among the phytochemicals identified by LC–MS/MS, suggesting that individual metabolites may contribute differently to the xenobiotic-sensing interactions computationally predicted for Rang Chuet. Among the compounds evaluated against PXR, α-spinasterol demonstrated the strongest predicted binding affinity (−10.13 kcal/mol), exceeding that of the co-crystallized agonist hyperforin (−9.53 kcal/mol) and the reference antagonist ketoconazole (−9.70 kcal/mol). This favourable binding energy, together with extensive hydrophobic interactions involving Leu206, Leu209, Leu240, Met243, Phe281, Phe429, and a stabilizing hydrogen bond with Lys210, suggests that α-spinasterol occupies the ligand-binding domain of PXR in a conformation consistent with a stable interaction; however, molecular docking cannot distinguish agonist from antagonist binding, so whether this interaction would result in receptor activation, inhibition, or partial modulation cannot be determined without functional assays.

PXR (NR1I2) is widely recognized as the master regulator of xenobiotic metabolism because activation of this nuclear receptor induces coordinated expression of Phase I cytochrome P450 enzymes, particularly CYP3A4, together with numerous Phase II conjugating enzymes including UDP-glucuronosyltransferases, glutathione S-transferases, sulfotransferases, and ATP-binding cassette transporters responsible for Phase III elimination [25,26,28,29]. This coordinated response enables organisms to rapidly metabolize and eliminate structurally diverse xenobiotics including pharmaceuticals, pesticides, plant toxins, dietary chemicals, and environmental contaminants. Consequently, activation of PXR represents one of the principal molecular mechanisms by which botanical medicines may enhance endogenous detoxification capacity.

The observation that α-spinasterol demonstrated the strongest predicted interaction with PXR is particularly interesting because this phytosterol was detected exclusively in *T. laurifolia*, the species that has historically received the greatest recognition within Thai traditional medicine and is included in Thailand’s National List of Essential Medicines for detoxification. Although α-spinasterol has primarily been investigated for its anti-inflammatory, antioxidant, neuroprotective, and hepatoprotective properties, relatively few studies have explored its potential influence on xenobiotic sensing pathways. The present docking results therefore generate the novel hypothesis that α-spinasterol may function as a natural PXR ligand; however, because docking cannot establish whether this interaction is agonistic or antagonistic, its net effect on detoxification capacity cannot be inferred without functional validation. Experimental validation using PXR reporter gene assays and CYP3A4 induction studies will be required to confirm this proposed mechanism.

Rosmarinic acid also demonstrated favourable interaction with PXR (−8.20 kcal/mol), exhibiting hydrogen bonding with Gln285 together with aromatic π-interactions involving Phe288 and Trp299. Rosmarinic acid is well recognized as one of the principal phenolic constituents of *T. laurifolia* and possesses potent antioxidant, anti-inflammatory, hepatoprotective, and cytoprotective properties [22]. Although previous investigations have attributed these activities primarily to direct scavenging of reactive oxygen species and modulation of inflammatory signalling pathways, the present docking results suggest that rosmarinic acid may additionally influence transcriptional regulation of detoxification pathways. Whether this interaction produces receptor activation or partial antagonism cannot be determined from docking alone, but the strong predicted affinity indicates that PXR should be considered a biologically relevant target for future mechanistic investigations.

In contrast, flavonoids including apigenin, quercetin, catechin, rutin, caffeic acid, and gallic acid exhibited moderate predicted affinities toward PXR. While these interactions were weaker than those observed for α-spinasterol, they may nevertheless contribute collectively to receptor modulation because herbal medicines characteristically contain numerous compounds capable of interacting simultaneously with multiple molecular targets. Such multi-component pharmacology is increasingly recognized as a defining characteristic of botanical medicines and may explain why complex herbal extracts frequently demonstrate biological activities that differ from those of isolated constituents.

The molecular docking studies against AhR revealed a distinctly different interaction profile. Among all phytochemicals examined, apigenin and catechin demonstrated the most favourable predicted binding affinity (−9.50 kcal/mol), followed by quercetin and caffeic acid (−8.70 and −8.00 kcal/mol). The docking pose of apigenin showed hydrogen bonding with Ser344 together with extensive hydrophobic interactions involving Phe293, Phe349, His289, and Leu351, producing a binding orientation closely resembling that of the co-crystallized agonist. These findings suggest that apigenin may function as a naturally occurring modulator of AhR signalling.

Historically, AhR was characterized primarily as the receptor responsible for mediating the toxicological effects of dioxins and related environmental pollutants. However, extensive research during the past two decades has fundamentally altered this perspective. AhR is now recognized as a multifunctional transcription factor involved in xenobiotic metabolism, immune regulation, intestinal barrier integrity, inflammation, oxidative stress responses, stem cell differentiation, and maintenance of tissue homeostasis [27,30,31]. Activation of AhR induces expression of CYP1A1, CYP1A2, CYP1B1, NAD(P)H quinone oxidoreductase-1, and numerous downstream genes involved in detoxification and cellular adaptation to chemical stress.

The strong predicted interaction between apigenin and AhR therefore provides an attractive mechanistic explanation linking the traditional detoxifying properties of Rang Chuet with contemporary molecular pharmacology. Rather than functioning solely as a direct antioxidant, apigenin may engage AhR-dependent signalling that influences cellular resilience against xenobiotic exposure; however, whether this interaction is agonistic or antagonistic, and therefore whether it enhances or suppresses detoxification capacity, cannot be determined from docking alone. This concept is consistent with previous reports demonstrating that dietary flavonoids modulate multiple nuclear receptors and transcription factors involved in oxidative stress, inflammation, and xenobiotic metabolism.

Quercetin also exhibited favourable binding to AhR, consistent with numerous previous reports demonstrating that quercetin influences AhR-dependent signalling pathways while simultaneously regulating NF-κB, Nrf2, MAPK, and PI3K/Akt signalling cascades. The presence of quercetin in every tissue examined from both botanical species further suggests that this flavonoid may provide a conserved molecular contribution to the putative xenobiotic-sensing interactions predicted across the different Rang Chuet species, pending functional validation. Similarly, catechin demonstrated appreciable binding affinity and has previously been shown to enhance antioxidant enzyme activity, reduce inflammatory cytokine production, and protect tissues against chemically induced oxidative injury.

Interestingly, α-spinasterol displayed mild predicted affinity for AhR despite exhibiting the strongest interaction with PXR. Meanwhile, apigenin showed more affinity favorable scores with both PXR and AhR. These complementary interaction profiles suggest that different phytochemicals within Rang Chuet may preferentially regulate distinct components of the detoxification network. Rather than relying upon a single active constituent acting through one receptor, the therapeutic activity of Rang Chuet appears more consistent with a systems pharmacology model in which multiple compounds simultaneously influence several interconnected signalling pathways governing xenobiotic metabolism, antioxidant defence, inflammation, and tissue protection.

An important aspect of xenobiotic biology is the extensive crosstalk that occurs between PXR, AhR, the Constitutive Androstane Receptor (CAR), and the Nuclear Factor Erythroid 2-Related Factor 2 (Nrf2) signalling pathway. These transcription factors function cooperatively to coordinate expression of metabolic enzymes, antioxidant proteins, inflammatory mediators, and transporter systems required for maintaining cellular homeostasis during exposure to endogenous and exogenous toxicants. Activation of one pathway frequently influences the activity of others, resulting in an integrated adaptive response rather than isolated receptor signalling. Consequently, the presence of multiple phytochemicals capable of interacting with different receptors may produce complementary or synergistic biological effects that cannot be predicted from individual compounds alone.

Collectively, the molecular docking results provide the first mechanistic evidence linking the traditional detoxification claims for Rang Chuet with modern concepts of xenobiotic sensing and transcriptional regulation. Exploratory docking suggested possible interactions of α-spinasterol with PXR and apigenin and catechin with AhR, generating hypotheses for future investigation of xenobiotic-sensing pathways. However, these predictions do not establish receptor activation or inhibition, changes in xenobiotic metabolism, or the detoxification efficacy or safety of the plant preparations. Although these computational predictions require validation using receptor activation assays, gene expression studies, enzyme activity measurements, and appropriate in vivo models, they provide a biologically plausible molecular framework that supports centuries of traditional use of Rang Chuet as a detoxifying herbal medicine and establishes a strong foundation for future mechanistic and translational investigations.

**Figure 5:**
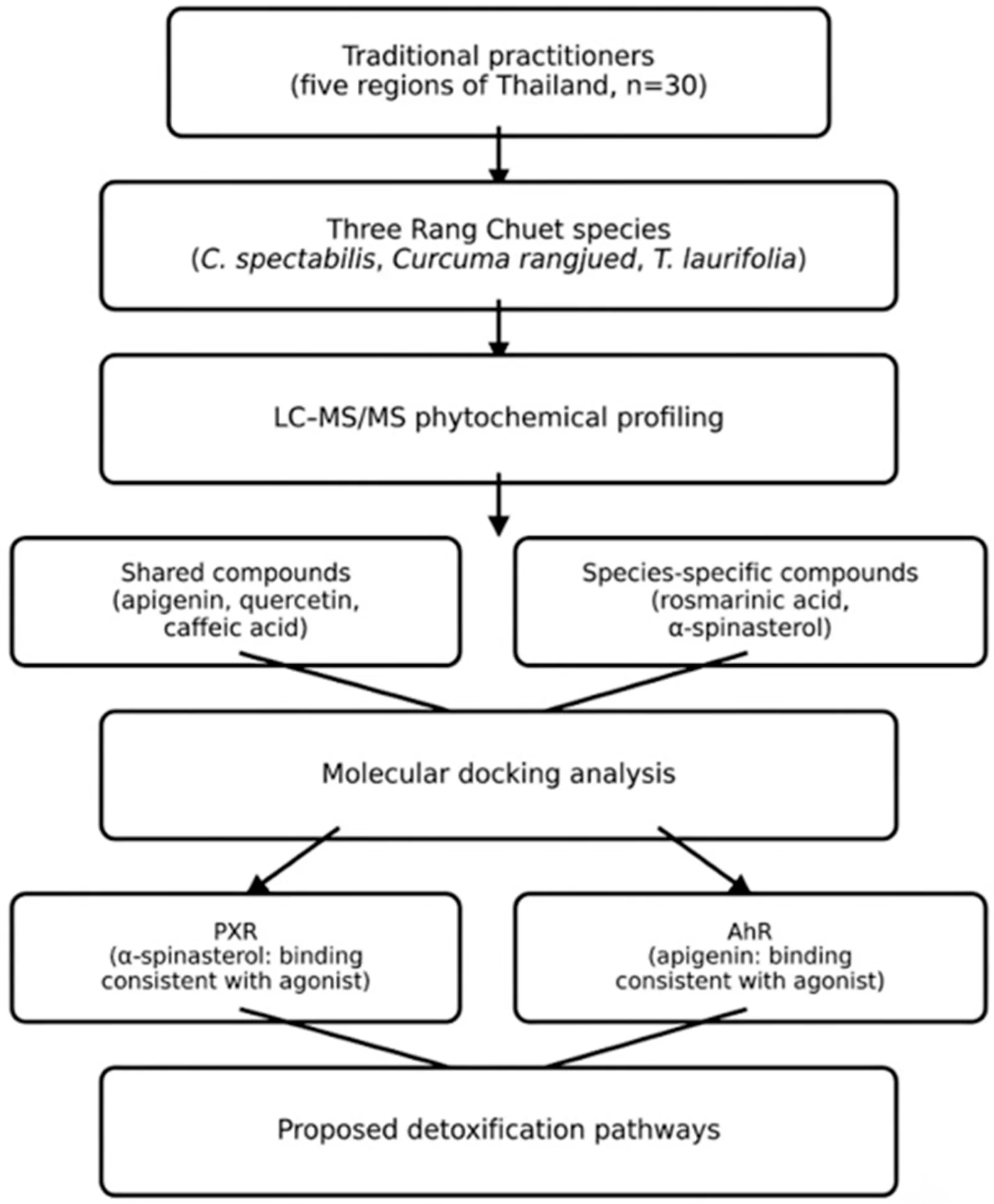
Conceptual summary linking traditional practitioner knowledge of Rang Chuet, botanical authentication of the three species collected under this name, LC–MS/MS phytochemical profiling showing shared and species-specific constituents, and molecular docking against PXR and AhR, illustrating the proposed mechanistic framework underlying the traditional detoxifying use of Rang Chuet.

Overall, the study began with an ethnopharmacological survey of 30 traditional practitioners from five regions of Thailand to identify the Rang Chuet species traditionally used for detoxification. Three species, *Crotalaria spectabilis* Roth, *Curcuma rangjued* Saensouk & Boonma, and *Thunbergia laurifolia* Lindl., were identified. Their phytochemical compositions were subsequently investigated using LC–MS/MS to characterize shared and species-specific compounds. Selected compounds, including apigenin, quercetin, caffeic acid, rosmarinic acid, and α-spinasterol, were further evaluated using molecular docking analysis against the pregnane X receptor (PXR) and aryl hydrocarbon receptor (AhR). The docking results were then used to propose potential molecular pathways underlying the detoxification-related effects of Rang Chuet species (Figure 5).

#### 3.3.1. Study Limitations

This study has several limitations that should be considered when interpreting its findings. The ethnopharmacological survey involved a relatively small sample of 30 traditional medicine practitioners, which may not fully capture the diversity of Rang Chuet use across Thailand. Molecular docking is a computational approach that generates testable hypotheses about ligand-receptor interactions but does not itself demonstrate receptor activation; no receptor activation assays or in vivo studies were performed to confirm the predicted interactions with PXR and AhR. Phytochemical characterisation by LC–MS/MS was performed for only two of the three identified Rang Chuet species. Furthermore, quantitative analysis could not be completed because the LC–MS/MS instrument was damaged, and quantitative data could not be retrieved. Therefore, the relative abundance of shared and species-specific constituents could not be determined. In addition, *Crotalaria spectabilis* Roth is a documented source of pyrrolizidine alkaloids, including monocrotaline, which are hepatotoxic and were not targeted by the LC–MS/MS or docking analyses performed here [32]. Toxicological characterisation of these constituents, together with functional validation of the predicted PXR and AhR interactions, is required before any conclusions can be drawn regarding the safety of *C. spectabilis*-derived Rang Chuet preparations. More broadly, receptor activation by a phytochemical does not by itself establish a beneficial detoxifying effect: PXR and AhR ligands can also promote bioactivation of pro-toxic substrates, so the docking results should be interpreted as evidence of xenobiotic-sensing potential rather than a demonstration of safety. Because molecular docking predicts binding pose and affinity but not functional outcome, it cannot distinguish whether a compound acts as a receptor agonist, antagonist, or partial modulator; the direction and magnitude of any effect on cytochrome P450-mediated detoxification therefore remain unknown for every interaction reported here. In the absence of compound quantification by LC–MS/MS, these findings should be regarded as hypothesis-generating and should not be used to recommend Rang Chuet, and particularly *C. spectabilis*, as a safe or effective detoxification remedy. Given these constraints, the present revision addresses the reviewers’ and Editor’s concerns primarily through reframing of the manuscript’s scope, title, and language rather than through new functional or in vitro assays, which could not be completed within the timeframe of this revision; functional validation of the predicted interactions (e.g., PXR/AhR reporter gene assays, CYP3A4/CYP1A1 induction studies) remains a priority for future work.

#### 3.3.2. Future Directions

Future studies should quantify the marker compounds identified here across a larger sample of plant material, compare phytochemical composition across seasons and growing regions, and characterise the third Rang Chuet species (*Curcuma rangjued* Saensouk & Boonma) by LC–MS/MS. Transcriptomic profiling and PXR reporter gene assays would help determine whether the predicted receptor interactions translate into functional changes in xenobiotic-metabolising gene expression, while targeted studies of cytochrome P450 induction would clarify the downstream metabolic consequences of PXR and AhR modulation by Rang Chuet constituents. Ultimately, animal toxicology and efficacy studies will be needed to translate these in silico and phytochemical findings into evidence-based recommendations for the traditional use of Rang Chuet as a detoxifying agent.

## 4. Materials and Methods

### 4.1. Ethnopharmacological Survey Methods

An ethnopharmacological survey was conducted among Thai herbal medicine practitioners from the 5 regions of Thailand. A total of 30 participants (6 from each region) with at least 10 years of clinical experience in traditional medicine were purposively recruited. Data were collected through structured questionnaires and face-to-face, in-depth interviews to obtain both quantitative and qualitative information regarding the traditional use of Rang Chuet for detoxification and the treatment of various diseases.

The questionnaire collected information on participants’ demographic characteristics, educational background, professional experience, areas of expertise, medicinal uses of Rang Chuet, methods of preparation, routes of administration, dosage, and plant species recognized under the vernacular name Rang Chuet. Qualitative interviews were conducted to verify questionnaire responses and to document traditional knowledge regarding the medicinal applications and botanical identity of the plants. Quantitative data were analyzed using descriptive statistics, including frequency, percentage, mean, and standard deviation. Qualitative data obtained from the interviews were analyzed descriptively and integrated with the survey findings to characterize the ethnopharmacological uses of Rang Chuet and to identify the plant species commonly employed in traditional practice.

Practitioners were eligible for inclusion if they had at least five years of documented clinical experience using Rang Chuet for detoxification and provided informed consent; individuals unable to describe specific preparation or administration practices were excluded. The interview questionnaire was developed from prior ethnobotanical instruments used in Thailand, reviewed by two independent ethnopharmacology researchers for content validity, and piloted with three practitioners before use. Interviewers received standardised training in semi-structured interview technique and plant specimen documentation prior to data collection. Practitioners were purposively sampled to represent all five regions of Thailand and a range of practice settings. The study protocol, including the informed consent procedure, was approved by the relevant institutional ethics review board prior to participant recruitment.

### 4.2. Sample Preparation and Extraction

Six plant samples comprising the leaves, roots, and stems of *Crotalaria spectabilis* Roth and *Thunbergia laurifolia* Lindl. were initially inspected for insect infestation and fungal contamination and washed twice with distilled water. The samples were oven-dried at 55 °C for 20 h, cooled to room temperature, and ground into a fine powder using a PHILIPS HR2061 blender (Koninklijke Philips N.V., Amsterdam, The Netherlands) at speed 3 for 5 min. The powdered samples were sieved to obtain particles smaller than 0.4 mm and stored in zip-lock bags in a desiccator until extraction. For maceration, 20 g of each powdered sample was placed in a 1000-mL flask and mixed with 500 mL of 45% ethanol (1:25, *w*/*v*). The flasks were sealed with Parafilm and agitated on a temperature-controlled orbital shaker at 37 °C and 150 rpm for 2 days. The extracts were then filtered through Whatman No. 1 filter paper. For Soxhlet extraction, each powdered sample was placed in an individual extraction thimble and extracted using a Soxhlet apparatus at 80–150 °C for 6 h. The resulting crude extracts were stored at 4 °C until further phytochemical analysis.

### 4.3. Phytochemical Analysis by LC–MS/MS

The phytochemical compositions of the crude *Crotalaria spectabilis* Roth and *Thunbergia laurifolia* Lindl. extracts were investigated using liquid chromatography-tandem mass spectrometry (LC–MS/MS). Approximately 10 mg of each lyophilized crude extract was accurately weighed and dissolved in 1 mL of methanol (1:1, *v*/*v*), vortexed for 30 s, and centrifuged at 10,000 rpm for 10 min. The resulting supernatant was filtered through a 0.22-µm PTFE syringe filter and transferred into LC–MS autosampler vials. Prepared samples were stored at 4 °C until analysis. Chromatographic separation was performed using an Acquity UPLC system (Waters Corporation, Milford, MA, USA) or Agilent 1290 Infinity II UHPLC system (Waldbronn, Germany) equipped with an Acquity UPLC BEH C18 (Waters Corporation, Milford, MA, USA) or Agilent Zorbax Eclipse Plus C18 column (Agilent Technologies, Santa Clara, CA, USA) (2.1 × 100 mm, 1.7 µm particle size) maintained at 40 °C. The mobile phases consisted of 0.1% formic acid in water (*v*/*v*) (solvent A) (SigmaAldrich, Steinheim, Germany) and 0.1%formic acid in acetonitrile (*v*/*v*) (solvent B) (SigmaAldrich, Steinheim, Germany) The gradient program was as follows: 5% B from 0.0 to 0.1 min, increased to 95% B at 10.0 min and maintained until 12.0 min, decreased to 5% B at 12.1 min, and maintained until 15.0 min. The flow rate was 0.3 mL/min, with an injection volume of 2 µL. Mass spectrometric detection was performed using either a Waters Xevo TQ-S micro triple quadrupole (Waters Corporation, Milford, MA, USA) or Thermo Scientific Q-Exactive Hybrid Quadrupole-Orbitrap mass spectrometer (Thermo Fisher Scientific, Bremen, Germany) equipped with an electrospray ionization (ESI) source operated in both positive and negative ion modes. The source parameters were set as follows: capillary voltage of 3.0 kV in positive mode and 2.5 kV in negative mode, cone voltage of 30 V, desolvation temperature of 450 °C, desolvation gas flow of 800 L/h, cone gas flow of 50 L/h, and nebulizer pressure of 4.0 bar. Data were acquired in full-scan mode over an *m*/*z* range of 50–1000, followed by data-dependent MS/MS (DDA) acquisition. Collision-induced dissociation (CID) was performed using a collision energy ramp of 15–45 V. The acquired LC–MS/MS data were subsequently used for the characterization and relative profiling of phytochemical constituents present in the plant extracts.

### 4.4. Molecular Docking Study Design

The molecular docking workflow consisted of receptor preparation, ligand preparation, docking simulation, and protein–ligand interaction analysis. Three-dimensional crystal structures of the target proteins associated with detoxification pathways were retrieved from the RCSB Protein Data Bank (RCSB PDB). Protein structures were prepared using BIOVIA Discovery Studio (Dassault Systèmes, San Diego, CA, USA) by removing crystallographic water molecules and co-crystallized ligands, adding hydrogen atoms, and optimizing the receptor structures for molecular docking.

Phytochemical constituents identified from the Rang Chuet extracts by LC–MS/MS were selected as ligands. Of the fifteen phytochemicals identified by LC–MS/MS, eight were carried forward for docking; the seven excluded compounds (chlorophyll a, chlorophyll b, pheophytin a, pheophorbide a, hydroxypheophytin a, lutein, and isoquercetin) are photosynthetic pigments and chlorophyll degradation products or glycosylated storage forms without established affinity for xenobiotic-sensing nuclear receptors, and were therefore excluded a priori in favour of the phenolic acids, flavonoids, and the phytosterol with documented receptor-relevant bioactivity. Their three-dimensional molecular structures were obtained from the PubChem database and subjected to geometry optimization prior to docking. Molecular docking simulations were performed using PyRx [33], employing the AutoDock Vina (version 1.2.5) algorithm to evaluate multiple ligand conformations and orientations within the receptor-binding pocket. The docking results were ranked according to the predicted binding free energy ($\Delta G_{\mathrm{bind}}$), with lower binding energy values indicating stronger binding affinity and greater complex stability. Protein–ligand interactions were subsequently analyzed using BIOVIA Discovery Studio to characterize hydrogen bonds, hydrophobic interactions, van der Waals interactions, and electrostatic interactions within the binding site. These interaction profiles were used to predict the binding mode of the phytochemicals and to elucidate their potential molecular mechanisms involved in detoxification.

Docking was performed with an exhaustiveness setting of 8 and a search-space grid of 25 × 25 × 25 Å centred on the respective ligand-binding domains of PXR and AhR. Docking protocol validity was confirmed by redocking each receptor’s native or co-crystallised reference ligand and calculating the root-mean-square deviation (RMSD) between the redocked and experimentally determined poses; poses with RMSD below 2.0 Å were considered acceptable. AutoDock Vina (within PyRx 0.8) was used for all docking runs, with ligand structures energy-minimised and protonated at physiological pH (7.4) prior to docking, and binding poses were ranked using the Vina scoring function.

## 5. Conclusions

This ethnopharmacological survey confirms that the vernacular name “Rang Chuet” is applied interchangeably by Thai traditional practitioners to at least three botanically distinct species, including *Crotalaria spectabilis* Roth, a documented source of hepatotoxic pyrrolizidine alkaloids. This botanical substitution represents an important public health concern that warrants clearer guidance for practitioners and consumers. Molecular docking suggested that phytochemicals characteristic of *Thunbergia laurifolia* Lindl., particularly α-spinasterol and apigenin, may interact with the xenobiotic-sensing receptors PXR and AhR; however, because docking cannot distinguish agonist from antagonist activity or establish functional receptor activation, and because LC–MS/MS quantification could not be completed, these interactions remain computational hypotheses rather than evidence of detoxification efficacy or safety. The findings should not be interpreted as validating any Rang Chuet species, and particularly not *C. spectabilis*, as a safe detoxification remedy. Rather, this work highlights the need for botanical authentication at the point of collection or sale, toxicological evaluation of *C. spectabilis*-derived preparations, and functional validation (e.g., PXR/AhR reporter gene assays) of the predicted receptor interactions before any pharmacological or clinical recommendations can be made.

## Figures and Tables

**Table 1:** Knowledge on the use of Rang Chuet herbal medicine.

Information	Number (Person)	Percentage (%)
Conditions treatable with Rang Chuet		
1 Antidote (detoxification)	30	100.0
Species of Rang Chuet used		
1 Rang Chuet tuber, Rang Chuet Ton, Rang Chuet vine	9	30.0
2 Rang Chuet tuber, Rang Chuet vine	4	13.3
3 Rang Chuet Ton, Rang Chuet vine	8	26.7
4 Rang Chuet vine	9	30.0
Wan Rang Chuet (part used)		
1 Rhizome	13	43.3
2 Not used	17	56.7
Rang Chuet Ton (part used)		
1 Stem, leaf, root	1	3.3
2 Stem, root	2	6.7
3 Stem	1	3.3
4 Leaf	4	13.3
5 Root	10	33.3
1 Stem, leaf, root	12	40.0
Rang Chuet Vine (part used)		
1 Vine, leaf, root	6	20.0
2 Vine, leaf	13	43.3
3 Vine, root	1	3.3
4 Leaf, root	4	13.3
5 Leaf	6	20.0
6 Not used	0	0.0
Rang Chuet Vine (Flower color)		
1 Purple	30	100.0
2 Purple, white	0	0
3 Purple, red	0	0
Preparation method for using Rang Chuet		
1 Grinding (ya fon)	1	3.3
2 Grinding, decoction	7	23.3
3 Grinding, fresh use	3	10.0
4 Decoction	8	26.7
5 Decoction, infusion	2	6.7
6 Decoction, fresh use	6	20.0
7 Infusion	3	10.0
Form of using Rang Chuet herbal medicine		
1 Single herb and herbal formula	13	43.3
2 Single herb only	17	56.7

**Table 2:** Identified phytochemicals in *Crotalaria spectabilis* and *Thunbergia laurifolia* extracts by LC-MS/MS. ✓: Indicates the presence of the compound.

Compound	*Crotalaria spectabilis*			*Thunbergia laurifolia*		
	Leaf	Stem	Root	Leaf	Stem	Root
α-spinasterol				✓		✓
Apigenin	✓	✓	✓	✓	✓	✓
Caffeic acid	✓	✓	✓	✓	✓	✓
Catechin	✓			✓		
Chlorophyll a		✓		✓		
Chlorophyll b						
Gallic acid	✓	✓	✓		✓	✓
Hydroxypheophytin a		✓	✓			✓
Isoquercetin	✓			✓		✓
Lutein		✓		✓		✓
Pheophorbide a	✓	✓		✓	✓	
Pheophytin a		✓			✓	
Quercetin	✓	✓	✓	✓	✓	✓
Rosmarinic acid				✓	✓	✓
Rutin	✓		✓	✓	✓	✓

**Table 3:** Predicted binding affinities of *Crotalaria spectabilis* and *Thunbergia laurifolia* compounds and control ligands with PXR (PDB ID 1M13) and AhR (PDB ID 8XS9).

Compound	PXR (PDB ID 1M13) Binding Affinity (kcal/mol) Seamdock-Vina	AhR (PDB ID 8XS9) Binding Affinity (kcal/mol) Seamdock-Vina
Hyperforin Co-crystallized ligand (PXR-agonist)	−9.53	-
Ketoconazole (PXR antagonist)	−9.7	-
β-Naphthoflavone Co-crystallized ligand (AhR-agonist)	-	−12.23
Ilantimod (BAY-2416964, AhR antagonist)	-	−4.93
α-spinasterol	−10.13	−5.4
Apigenin	−8.06	−9.5
Caffeic acid	−6.06	−8
Catechin	−7.83	−9.5
Gallic acid	−5.93	−6.9
Quercetin	−7.86	−8.7
Rosmarinic acid	−8.2	−7.3
Rutin	−9.7	−4.83
Monocrotaline (distinctive compound in seed of Crotalaria species)	−7.9	−5.26

Note: Molecular docking was performed using SEAMDOCK. Grid centers and sizes were set to (3, 8, 0) Å and (32, 34, 35) Å for PXR (1M13), and (−36, −8, −17) Å and (18, 22, 20) Å for AhR (8XS9), respectively. Hyperforin and β-naphthoflavone were used as reference ligands for PXR and AhR, respectively.

## Data Availability

The data presented in this study are available on request from the corresponding author due to ethical reasons.

## Supplementary Materials

The following supporting information can be downloaded at: https://www.mdpi.com/article/10.3390/ijms27188389/s1.

## Abbreviations

The following abbreviations are used in this manuscript:

ABC	ATP-binding cassette
ABCB1	P-glycoprotein
ADH	Alcohol dehydrogenase
ALDH	Aldehyde dehydrogenase
CAT	Catalase
CYP1A2	Cytochrome P450 1A2
CYP2E1	Cytochrome P450 2E1
FMOs	Flavin-containing monooxygenases
GSTs	Glutathione S-transferases
GPx	Glutathione peroxidase
MRPs	Multidrug resistance-associated proteins
NATs	N-acetyltransferases
OATs	Organic anion transporters
OCTs	Organic cation transporters
SULTs	Sulfotransferases
UGTs	UDP-glucuronosyltransferases
