# Supplementary material for: Botanical Substitution in Traditional ‘Rang Chuet’ Preparations: Distinguishing the Potential Xenobiotic Modulation of Thunbergia laurifolia Lindl. from the Hepatotoxic Risks of Crotalaria spectabilis Roth
Source: Int J Mol Sci. 2026 Sep 20;27(18):8389. doi: 10.3390/ijms27188389 (PMC13607412; doi:10.3390/ijms27188389)
Supplement: Supplementary file 1 [file ijms-27-08389-s001.zip › ijms-4503174-supplementary.pdf]

Supplement Table S1. Demographic characteristics of folk healers (N=30).

Characteristic	Number (persons)	Percentage (%)	Notes
Sex			
Male	25	83.3	
Female	5	16.7	
Age (years)	-	-	Mean ± SD = 68.27 ± 15.55; Max = 92.0, Min = 42.0
Education level			
Below bachelor's degree	26	86.7	
Bachelor's degree	4	13.3	
Occupation			
Traditional healer	12	40.0	
Agriculture	12	40.0	
Trade/Private business	3	10.0	
Government or state enterprise employee	2	6.7	
Monk	1	3.3	

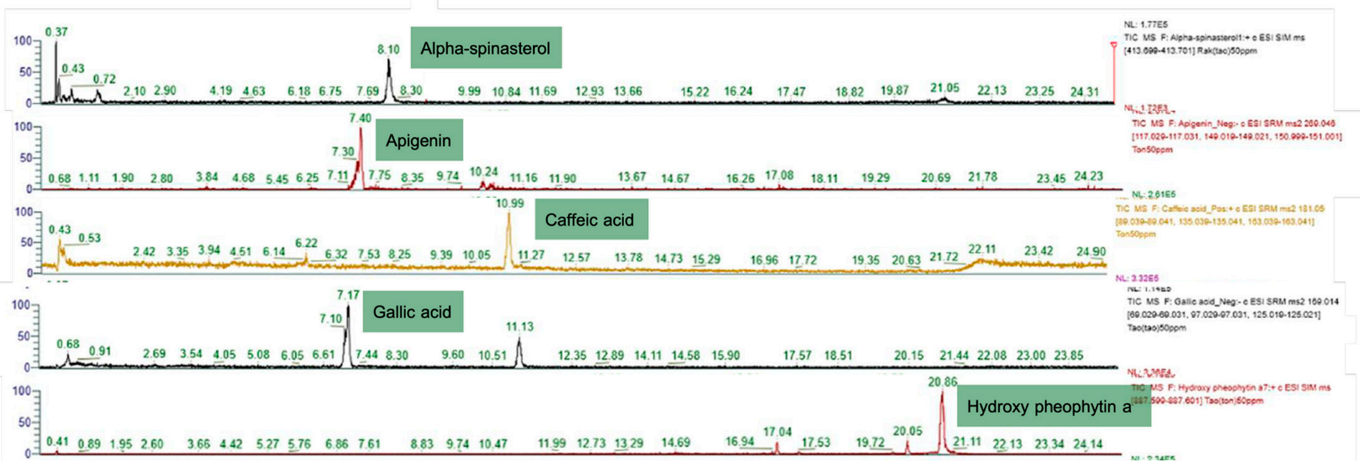

Figure S1a: Representative Extracted Ion Chromatograms (EICs) of Alpha-spinasterol, Apigenin, Caffeic acid, Gallic acid, and Hydroxypheophytin a from Crotalaria spectabilis and Thunbergia laurifolia extracts

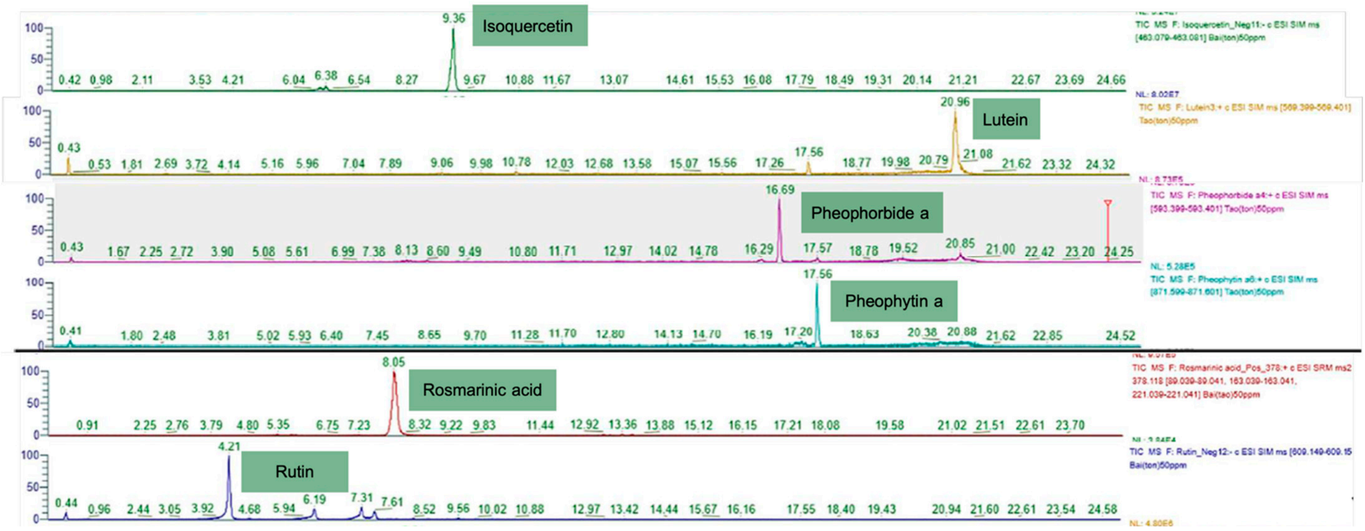

Figure S1b : Representative Extracted Ion Chromatograms (EICs) of Isoquercetin, Lu-tein, Pheophorbide a, Pheophytin a, Rosmarinic acid, and Rutin from Crotalaria spectabilis and Thunbergia laurifolia extracts.
